# Supplementary material for: Targeting the Gastrin-Releasing Peptide Receptor (GRP-R) in Cancer Therapy: Development of Bombesin-Based Peptide–Drug Conjugates
Source: Int J Mol Sci. 2023 Feb 8;24(4):3400. doi: 10.3390/ijms24043400 (PMC9967152; doi:10.3390/ijms24043400)
Supplement: Supplementary file 1 [file ijms-24-03400-s001.zip › ijms-2144577-supplementary.pdf]

International Journal of
*Molecular Sciences*

MDPI

*Article: Supplementary material*

# Targeting the Gastrin Releasing Peptide Receptor (GRP-R) in Cancer Therapy: Development of Bombesin Based Peptide-Drug Conjugates

**Jacopo Gomena [1,2], Balázs Vári [3], Rita Oláh-Szabó [4], Beáta Biri-Kovács [1,2], Szilvia Bősze [2], Adina Borbély [1,5], Ádám Soós [6], Ivan Ranđelović [3,7], József Tóvári [3] and Gábor Mező [1,2,*]**

1 Institute of Chemistry, Faculty of Science, Eötvös Loránd University, 1117 Budapest, Hungary
2 ELKH-ELTE Research Group of Peptide Chemistry, 1117 Budapest, Hungary
3 Department of Experimental Pharmacology, National Institute of Oncology, 1122 Budapest, Hungary
4 Department of Genetics, Cell and Immunobiology, Semmelweis University, 1089 Budapest, Hungary
5 MTA-ELTE Lendület Ion Mobility Mass Spectrometry Research Group, 1117 Budapest, Hungary
6 Department of Anatomy, Histology and Embryology, Semmelweis University, 1085 Budapest, Hungary
7 KINETO Lab Ltd., 1037 Budapest, Hungary
* Correspondence: gabor.mezo@ttk.elte.hu

**Table of Content**

RP-HPLC chromatogram and ESI-HRMS spectrum of **G1** 2
RP-HPLC chromatogram and ESI-HRMS spectrum of **G2** 2
RP-HPLC chromatogram and ESI-HRMS spectrum of **G3** 2
RP-HPLC chromatogram and ESI-HRMS spectrum of **G4** 3
RP-HPLC chromatogram and ESI-HRMS spectrum of **G5** 3
RP-HPLC chromatogram and ESI-HRMS spectrum of **L1** 3
RP-HPLC chromatogram and ESI-HRMS spectrum of **L2** 4
RP-HPLC chromatogram and ESI-HRMS spectrum of **L3** 4
RP-HPLC chromatogram and ESI-HRMS spectrum of **L4** 4
RP-HPLC chromatogram and ESI-HRMS spectrum of **L5** 5
RP-HPLC chromatogram and ESI-HRMS spectrum of **L6** 5
RP-HPLC chromatogram and ESI-HRMS spectrum of **FP1** 5
RP-HPLC chromatogram and ESI-HRMS spectrum of **FP2** 6
Western Blot analysis of GRP-R protein level expression on
MDA-MB-231, MDA-MB-453 and PC-3 human cancer cell lines 6
Dose-response curves of the PDCs and Dau on human fibroblasts MRC-5 7
Cellular uptake of the PDCs on MDA-MB-231, MDA-MB-453 and PC-3
human cancer cell lines (flow cytometry measurements) 7
Stability of the PDCs in cell culture medium 8
Stability of **L1**, **L5** and **L6** in mouse plasma (Extracted Ion Chromatograms) 8
Metabolites produced by **L1**, **L5** and **L6**
in presence of lysosomal rat liver homogenate 9
Mice weight change during the tumour growth inhibition experiment 10

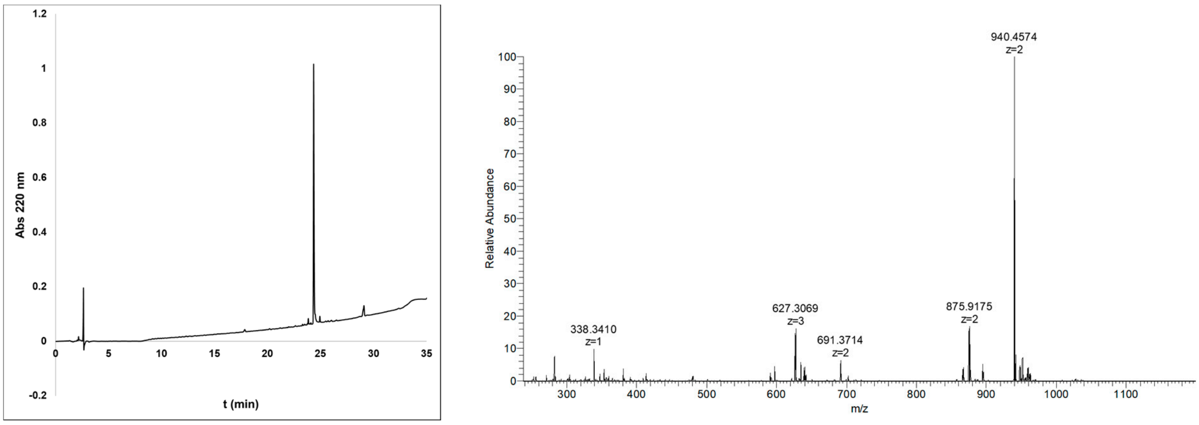


**Figure S1.** RP-HPLC chromatogram and ESI-HRMS spectrum of **G1**. *Rt* = 24.35 min. $MW_{meas}$ / $MW_{cal}$: 1878.8988 / 1879.1050 g/mol. A signal diminished by about 129 g/mol from the expected MW is observed in all the MS spectra of the conjugates due to the cleavage of the Dau glycosidic bond during the MS measurement [1].

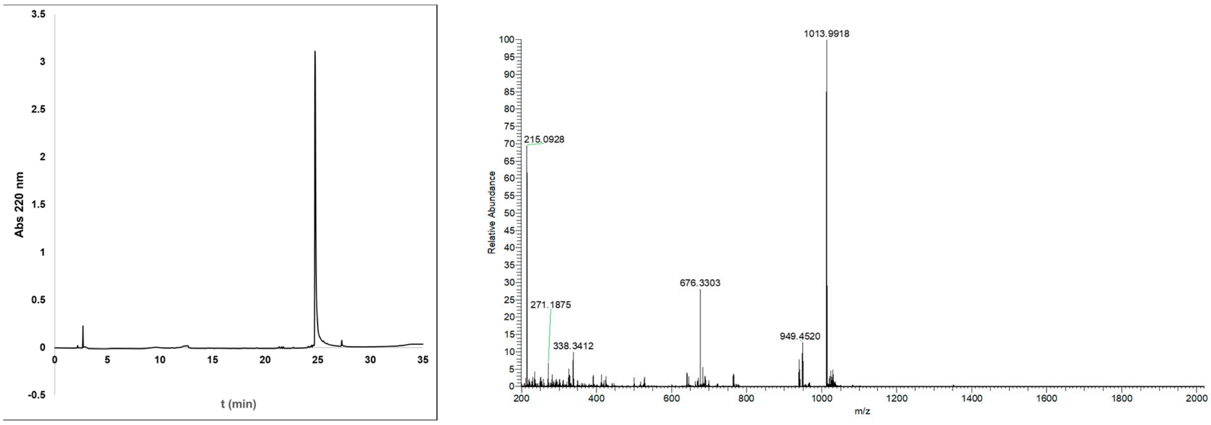


**Figure S2.** RP-HPLC chromatogram and ESI-HRMS spectrum of **G2**. *Rt* = 24.75 min. $MW_{meas}$ / $MW_{cal}$: 2025.9836 / 2026.2820 g/mol.

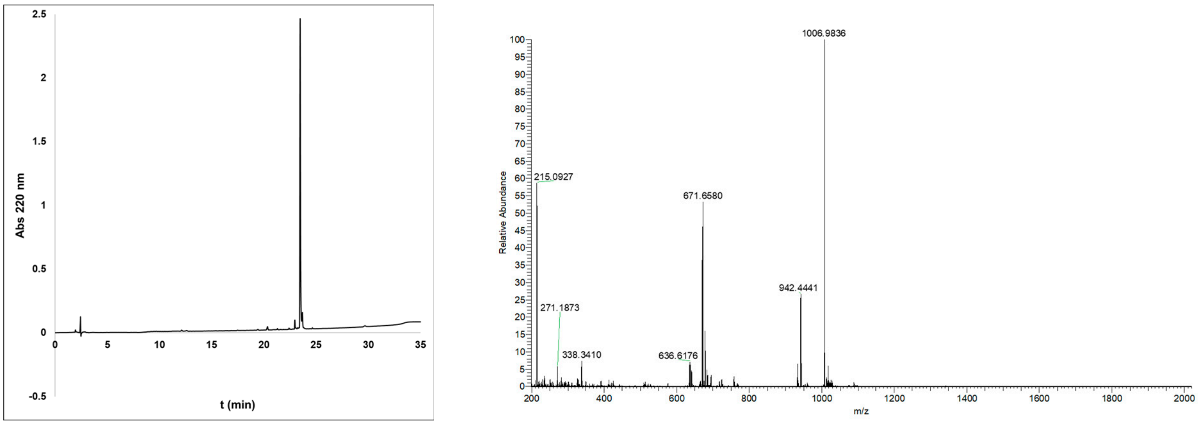


**Figure S3.** RP-HPLC chromatogram and ESI-HRMS spectrum of **G3**. *Rt* = 24.45 min. $MW_{meas}$ / $MW_{cal}$: 2011.9672 / 2012.2550 g/mol.

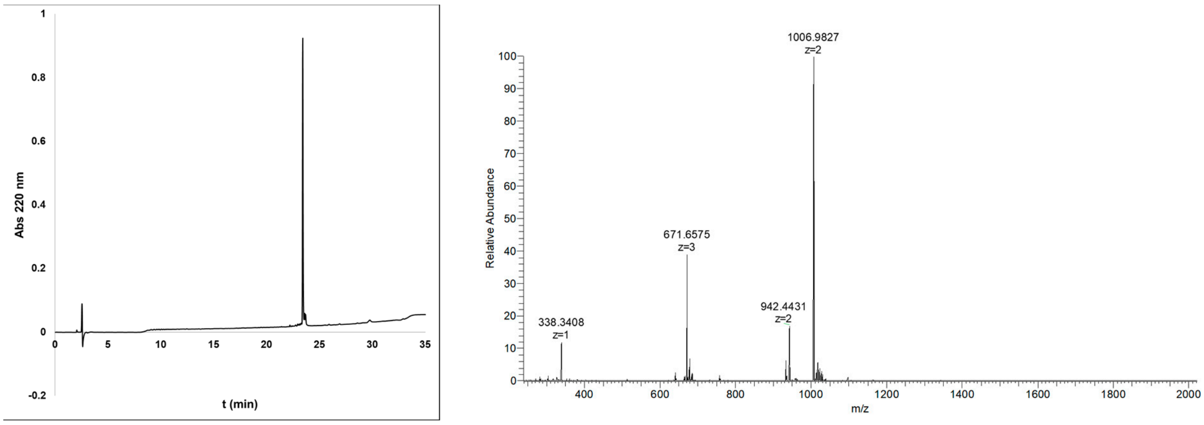


**Figure S4.** RP-HPLC chromatogram and ESI-HRMS spectrum of **G4**. *Rt* = 23.42 min. $MW_{meas}$ / $MW_{cal}$: 2011.9494 / 2012.2550 g/mol.

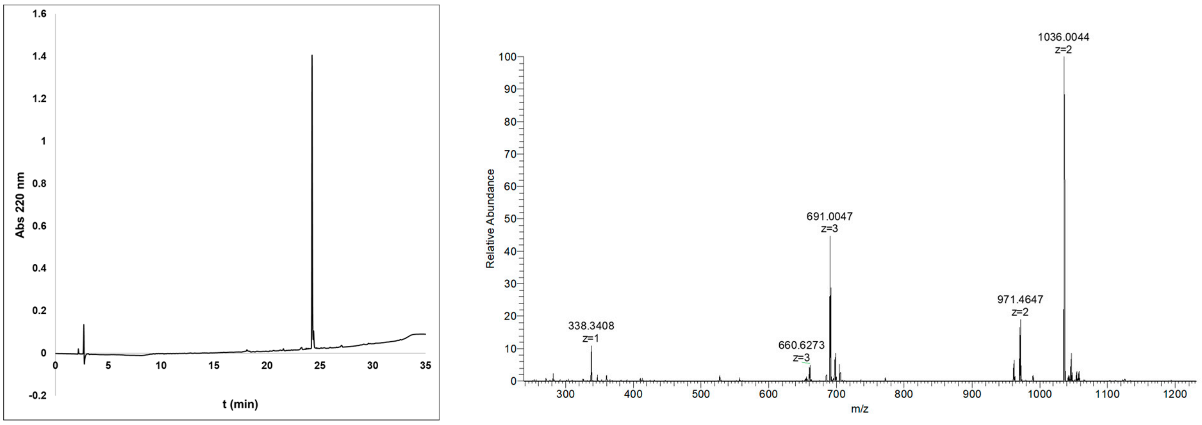


**Figure S5.** RP-HPLC chromatogram and ESI-HRMS spectrum of **G5**. *Rt* = 24.25 min. $MW_{meas}$ / $MW_{cal}$: 2069.9928 / 2070.3350 g/mol.

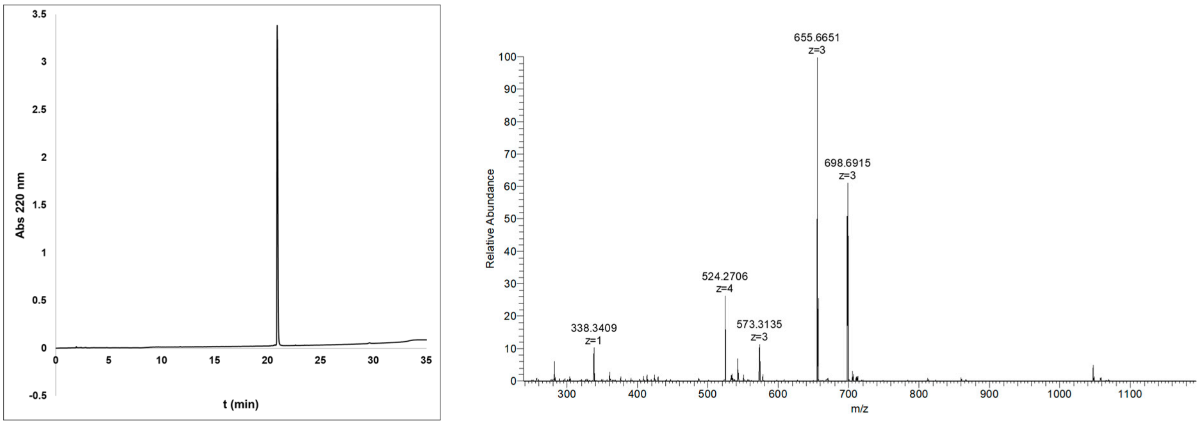


**Figure S6.** RP-HPLC chromatogram and ESI-HRMS spectrum of **L1**. *Rt* = 20.93 min. $MW_{meas}$ / $MW_{cal}$: 2093.0528 / 2093.3780 g/mol.

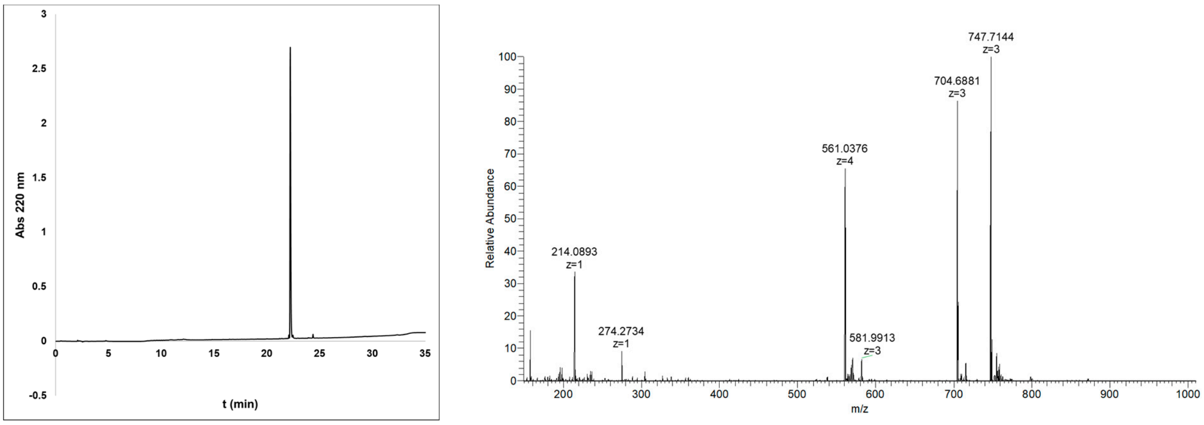


**Figure S7.** RP-HPLC chromatogram and ESI-HRMS spectrum of **L2**. *Rt* = 22.22 min. $MW_{meas}$ / $MW_{cal}$: 2240.1192 / 2240.5550 g/mol.

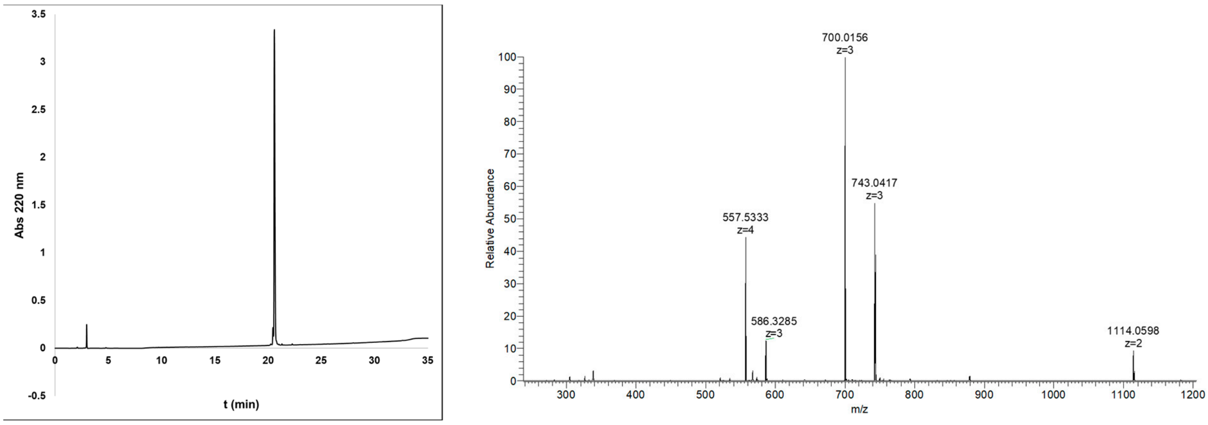


**Figure S8.** RP-HPLC chromatogram and ESI-HRMS spectrum of **L3**. *Rt* = 21.23 min. $MW_{meas}$ / $MW_{cal}$: 2226.1036 / 2226.5280 g/mol.

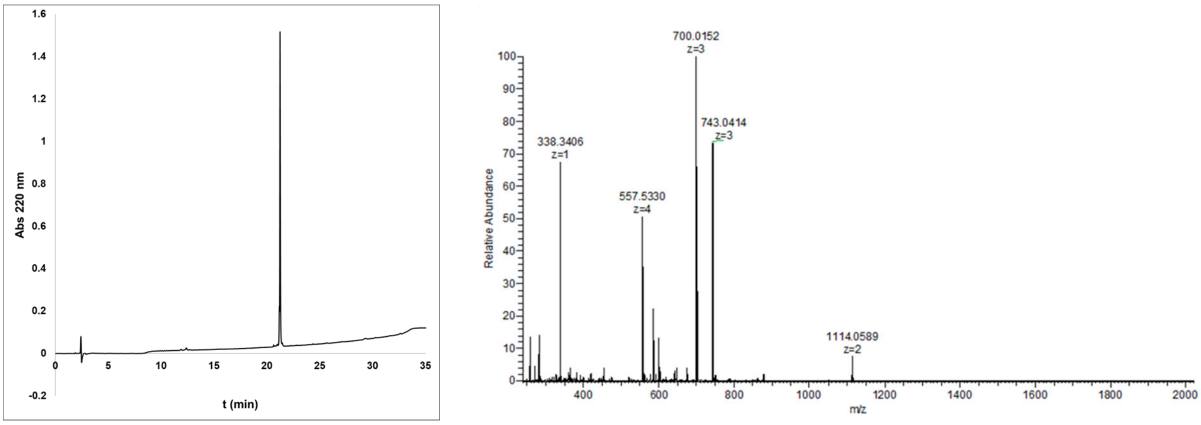


**Figure S9.** RP-HPLC chromatogram and ESI-HRMS spectrum of **L4**. *Rt* = 20.58 min. $MW_{meas}$ / $MW_{cal}$: 2226.1018 / 2226.5280 g/mol.

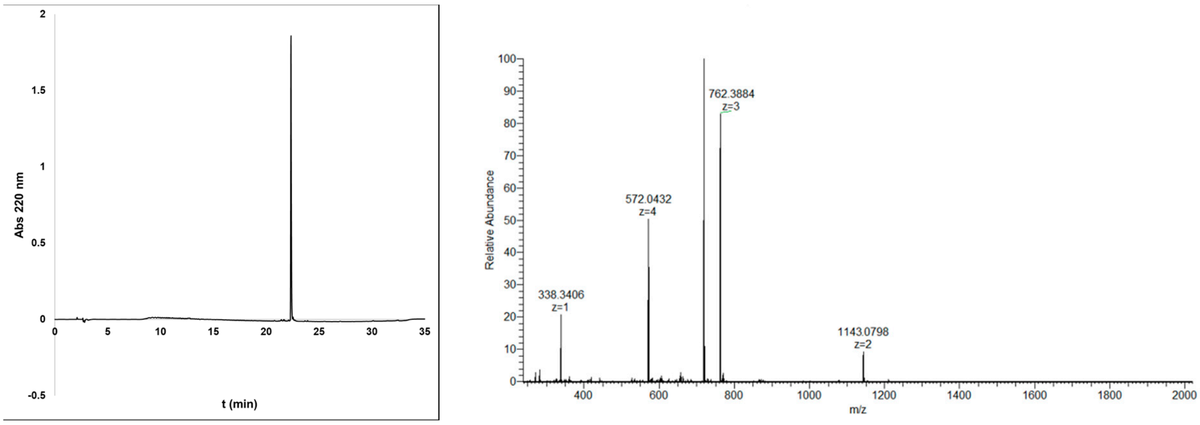


**Figure S10.** RP-HPLC chromatogram and ESI-HRMS spectrum of **L5**. *Rt* = 22.35 min. $MW_{meas}$ / $MW_{cal}$: 2284.1462 / 2284.6080 g/mol.

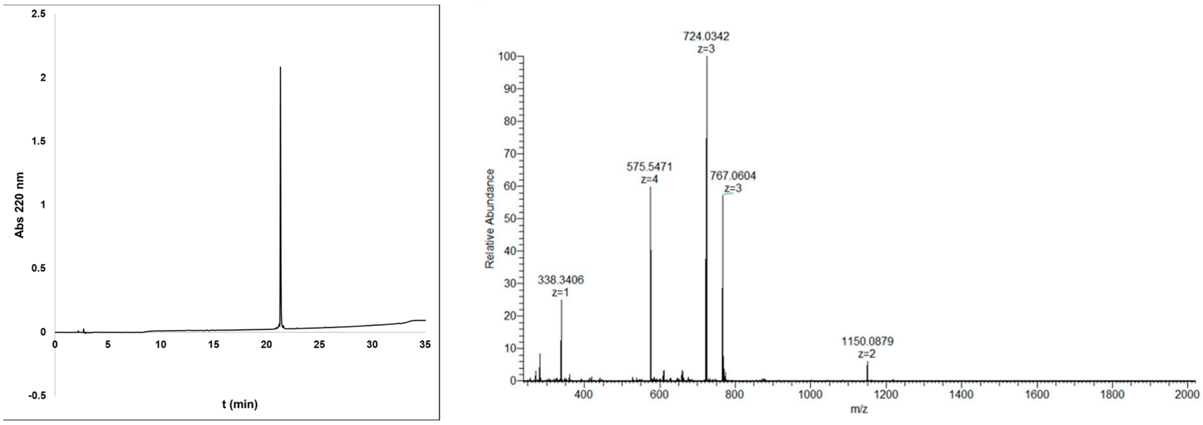


**Figure S11.** RP-HPLC chromatogram and ESI-HRMS spectrum of **L6**. *Rt* = 21.30 min. $MW_{meas}$ / $MW_{cal}$: 2298.1598 / 2298.6350 g/mol.

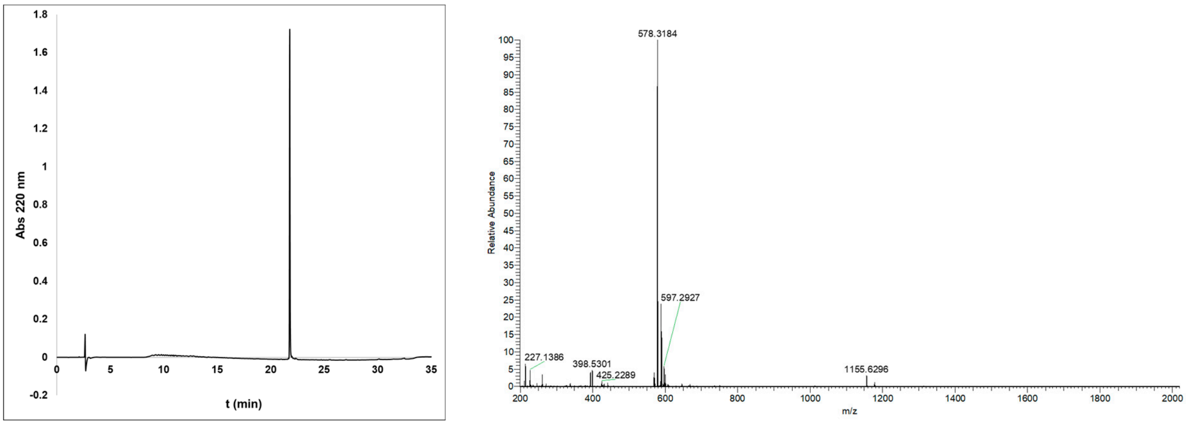


**Figure S12.** RP-HPLC chromatogram and ESI-HRMS spectrum of **FP1**. *Rt* = 21.76 min. $MW_{meas}$ / $MW_{cal}$: 1155.6368 / 1155.3690 g/mol.

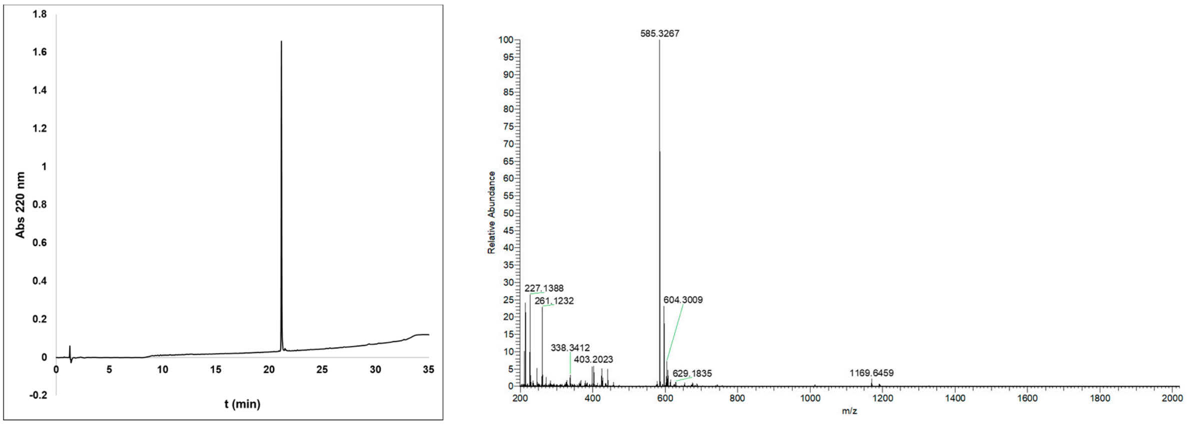


**Figure S13.** RP-HPLC chromatogram and ESI-HRMS spectrum of **FP2**. *Rt* = 21.17 min. $MW_{meas}$ / $MW_{cal}$: 1168.6534 / 1169.3960 g/mol.

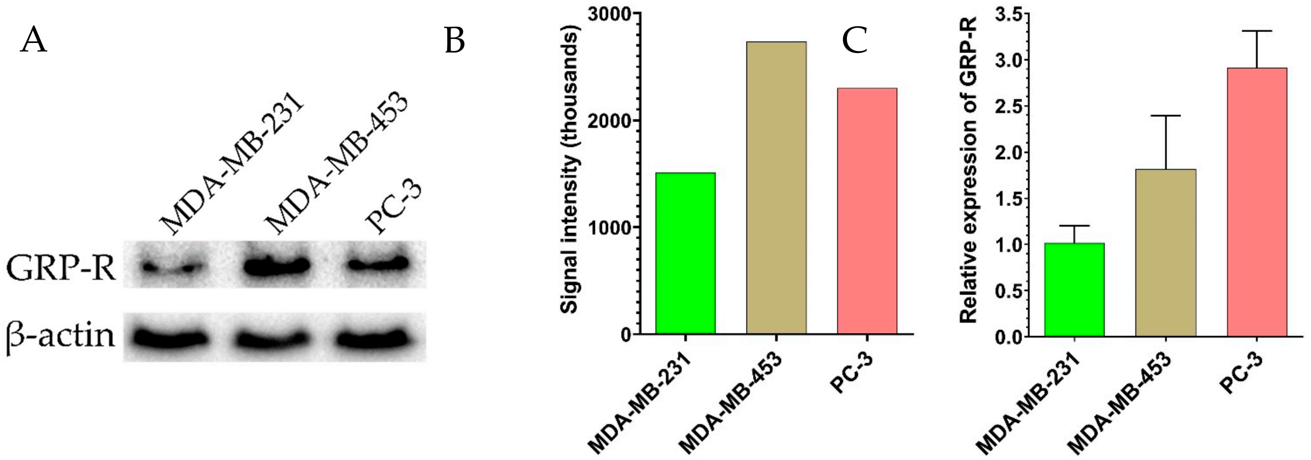


**Figure S14.** GRP-R expression levels in human cancer cell lines PC-3 (prostate), MDA-MB-453 and MDA-MB-231 (breast). (**A**) Western Blot and (**B**) quantification of the total GRP-R protein expression. The expression of β-actin was assessed as loading control and used for normalisation of the GRP-R content. (**C**) mRNA relative expression of GRP-R, normalised to the lowest expressing cell line, MDA-MB-231 (mean ± SD, n = 3).

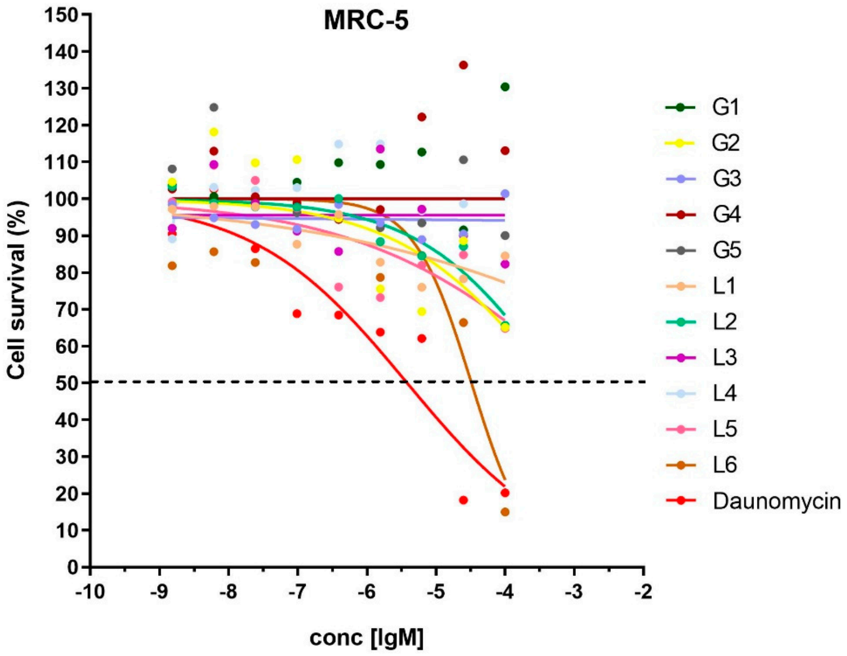


**Figure S15.** Dose-response curves of the PDCs and Dau on MRC-5 human fibroblast cell line. Only Dau reveals toxicity on the cells.

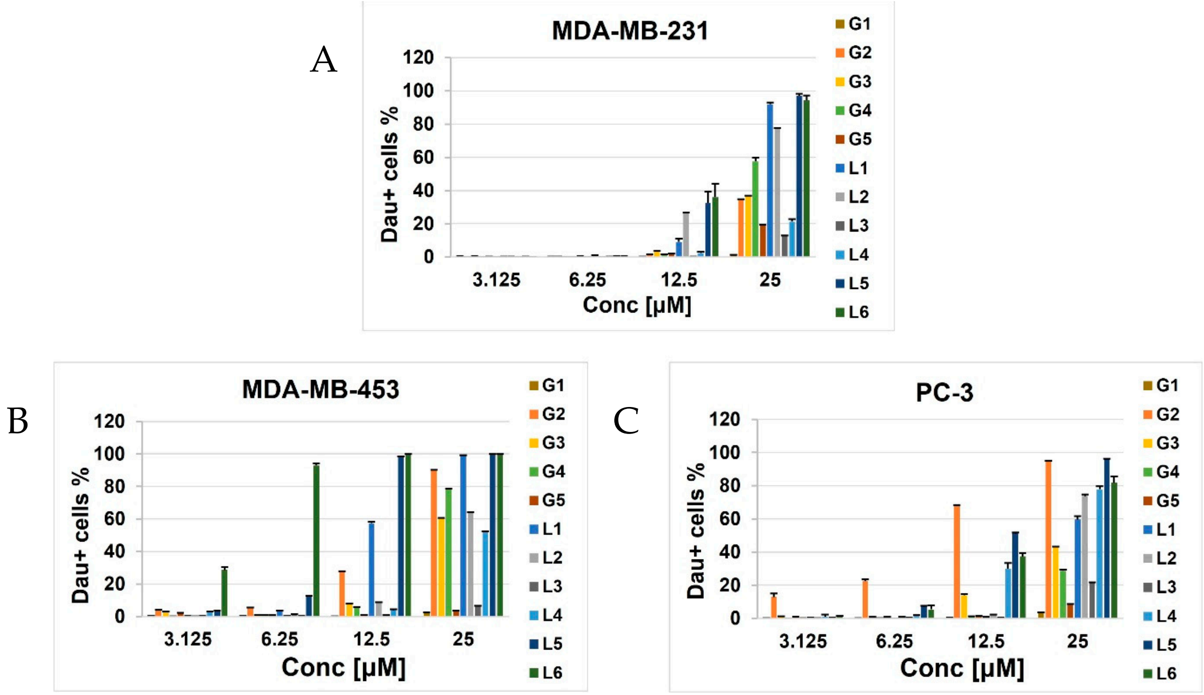


**Figure S16.** Flow cytometry measurements for the uptake of all the PDCs on MDA-MB-231 (**A**), MDA-MB-453 (**B**) and PC-3 (**C**) human cancer cell lines. The percentage of cells containing the Dau-conjugates was evaluated on four concentrations (%, mean ± SD, n= 2). Given that above 12.5 µM the internalisation may not only be receptor-mediated, this concentration was considered to compare the uptake of the bioconjugates.

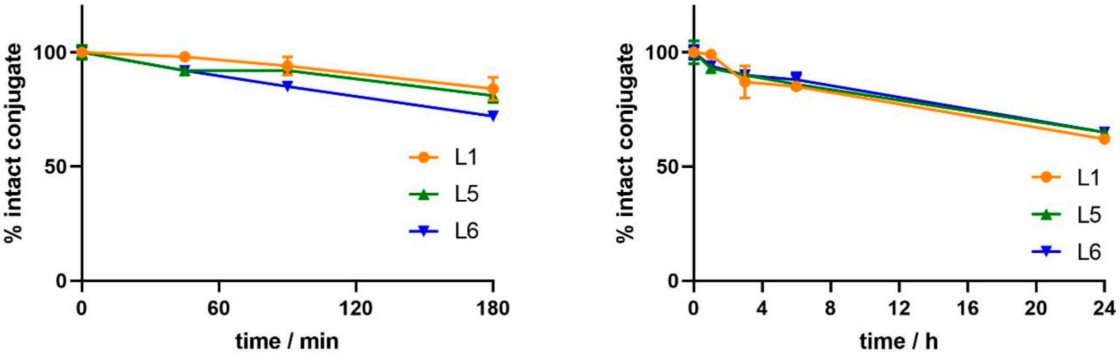


**Figure S17.** Stability of **L1**, **L5** and **L6** in serum free (**left**) and 2.5 % FBS containing (**right**) DMEM (%, mean ± SD, n = 2). The assays were performed over 3 h and 24 h, respectively, to monitor any possible loss of conjugate that could affect the cellular uptake and MTT assays, respectively.

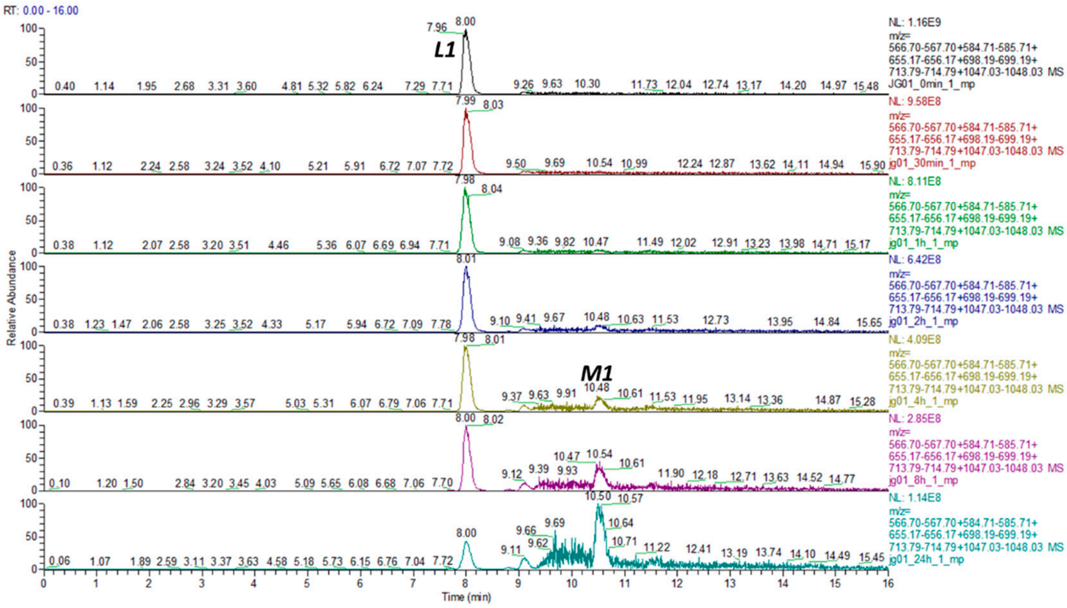


**Figure S18.** Plasma stability of **L1**. Mass Spectrometry Extracted Ion Chromatograms (EIC) at the collection time points (0 min, 30 min, 1 h, 2 h, 4 h, 8 h, 24 h) for **L1** and the cytotoxic metabolite **Dau=Aoa-Leu-OH** (**M1**).

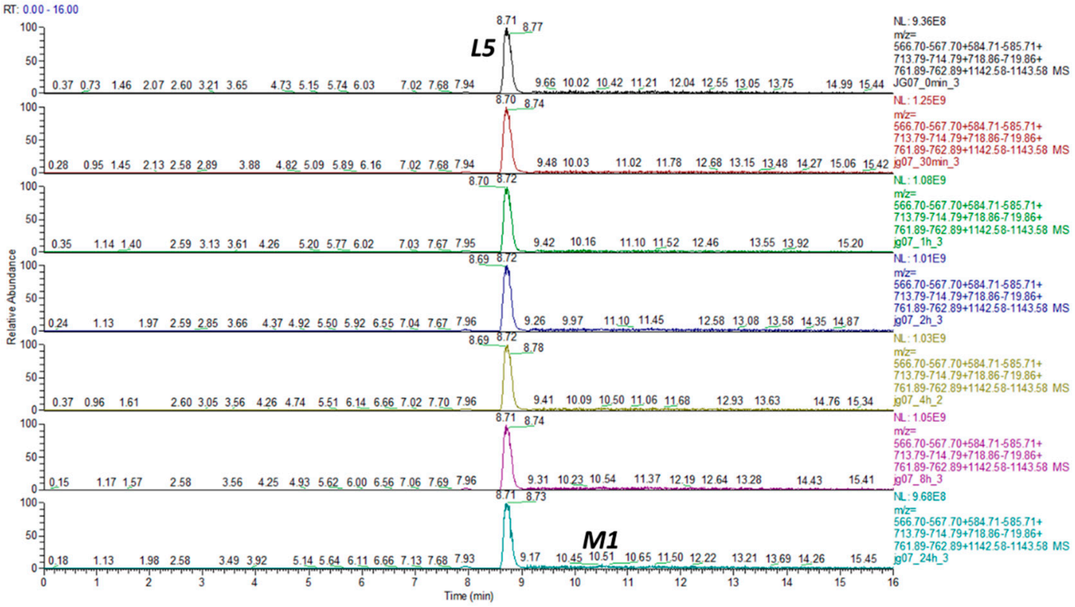


**Figure S19.** Plasma stability of **L5**. Mass Spectrometry Extracted Ion Chromatograms (EIC) at the collection time points (0 min, 30 min, 1 h, 2 h, 4 h, 8 h, 24 h) for **L5** and the cytotoxic metabolite **Dau=Aoa-Leu-OH** (**M1**).

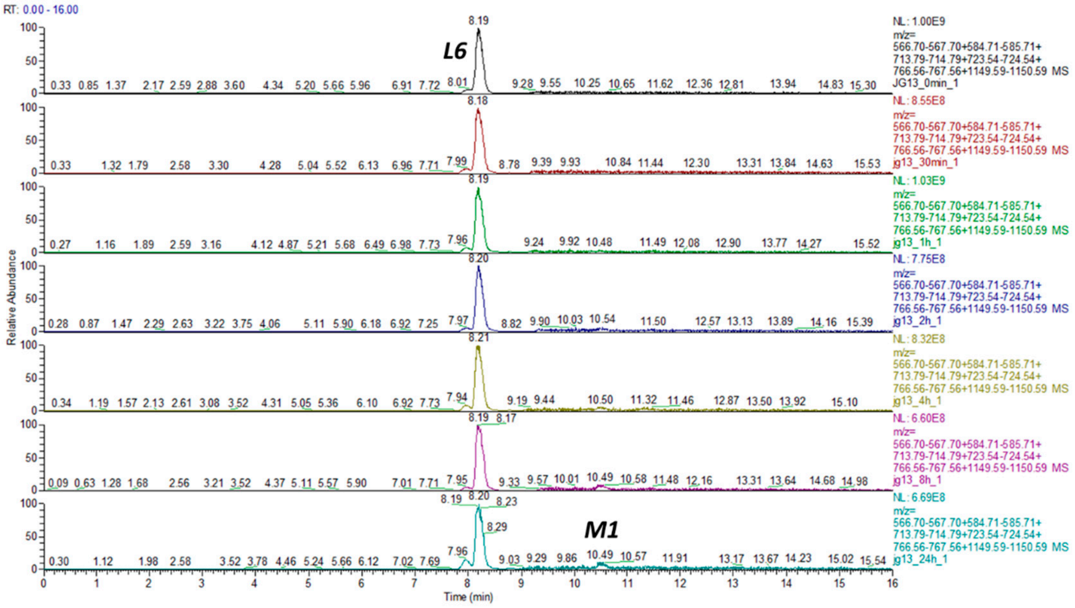


**Figure S20.** Plasma stability of **L6**. Mass Spectrometry Extracted Ion Chromatograms (EIC) at the collection time points (0 min, 30 min, 1 h, 2 h, 4 h, 8 h, 24 h) for **L6** and the cytotoxic metabolite **Dau=Aoa-Leu-OH** (**M1**).

**Table S1.** Metabolites obtained by the degradation of **L1**, **L3**, **L5** and **L6** in rat liver lysosomal homogenate. Only the metabolites giving clearly visible peaks in the chromatograms are reported.

| Code | Compound | Metabolite | $MW_{meas}$ / $MW_{cal}$ (g/mol) |
|---|---|---|---|
| L1 | (Dau=Aoa)-LRRY-BBN(7-14)[Nle$^{14}$] | Dau=Aoa-LRR-OH | 1025.49 / 1026.12 |
| | | Dau=Aoa-LR-OH | 869.39 / 869.93 |
| | | Dau=Aoa-L-OH | 713.29 / 713.74 |
| L3 | (Dau=Aoa)-LRRY-BBN[D-Phe$^6$, β-Ala$^{11}$, Aib$^{13}$, Nle$^{14}$] | $H_2N$-RY-fQWAV-β-Ala-H-Aib-Nle-$NH_2$ | 1374.74 / 1374.62 |
| | | $H_2N$-Y-fQWAV-β-Ala-H-Aib-Nle-$NH_2$ | 1218.64 / 1218.43 |
| | | $H_2N$-RY-fQW-OH | 799.39 / 798.90 |
| | | Dau=Aoa-LR-OH | 869.39 / 869.93 |
| | | Dau=Aoa-L-OH | 713.29 / 713.74 |
| L5 | (Dau=Aoa)-LRRY-BBN[D-Phe$^6$, Sta$^{13}$, Leu$^{14}$] | $H_2N$-RY-fQWAVGH-Sta-L-$NH_2$ | 1431.80 / 1432.70 |
| | | $H_2N$-VGH-Sta-L-$NH_2$ | 580.38 / 580.73 |
| | | $H_2N$-GH-Sta-L-$NH_2$ | 481.31 / 481.60 |
| | | $H_2N$-RY-fQW-OH | 798.39 / 798.90 |
| | | $H_2N$-RYfQ-OH | 612.31 / 612.69 |
| | | Dau=Aoa-LR-OH | 869.39 / 869.93 |
| | | Dau=Aoa-L-OH | 713.29 / 713.74 |
| L6 | (Dau=Aoa)-LRRY-BBN[D-Phe$^6$, β-Ala$^{11}$, Sta$^{13}$, Nle$^{14}$] | $H_2N$-RY-fQWAV-βAla-H-Sta-Nle-$NH_2$ | 1445.80 / 1446.72 |
| | | $H_2N$-Y-fQWAV-βAla-H-Sta-Nle-$NH_2$ | 1290.68 / 1290.54 |
| | | $H_2N$-LRRY-fQWAV-βAla-H-$NH_2$ | 1446.78 / 1446.68 |
| | | $H_2N$-RY-fQW-OH | 798.39 / 798.90 |
| | | Dau=Aoa-LR-OH | 869.39 / 869.93 |
| | | Dau=Aoa-L-OH | 713.29 / 713.74 |

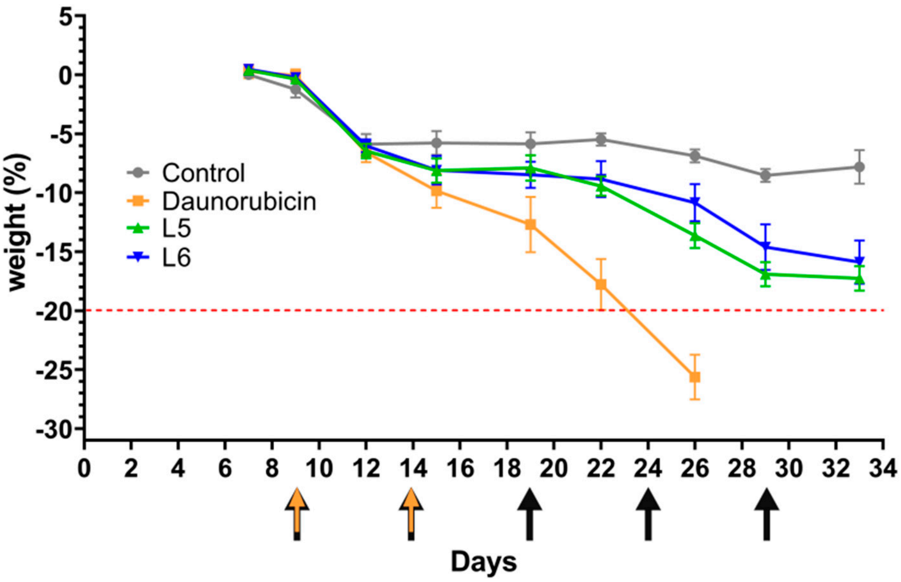


**Figure S21.** Mouse weight variation during the tumour growth inhibition experiment (%, mean ± SEM, n = 8). Administration regime: 0.9% saline (control group, black arrows), Dau (1 mg/kg, orange arrows) and PDCs (10 mg/kg calculated on Dau content, black arrows), every 5th day starting from Day 9.

## References

1. Pethő, L.; Mező, G.; Schlosser, G. Overcharging Effect in Electrospray Ionization Mass Spectra of Daunomycin-Tuftsin Bioconjugates. *Molecules* **2019**, *24*, 2981. https://doi.org/10.3390/molecules24162981
